# Supplementary material for: Kinetic study of catalytic CO2 hydration by metal-substituted biomimetic carbonic anhydrase model complexes
Source: R Soc Open Sci. 2019 Aug 7;6(8):190407. doi: 10.1098/rsos.190407 (PMC6731748; doi:10.1098/rsos.190407)
Supplement: Supplementary Figures and Tables [file rsos190407supp1.docx]

**Table S1. Determination of X-ray structure of [(TPA)Ni(OH_2_)](SO_4_)**

| Identification code | 0130b-2 |
| --- | --- |
| Empirical formula | C20 H28 N4 Ni O7 S |
| Formula weight | 527.23 |
| Temperature | 120 (2) K |
| Wavelength | 0.71073 Å |
| Crystal system | Orthorhombic |
| Space group | *Pca*2 (1) |
| Unit cell dimensions | *a* = 27.666 (4) Å |
|  | *b* = 8.5798 (13) Å |
|  | *c* = 9.6808 (14) Å |
| Volume | 2297.9 (6) Å3 |
| *Z* | 4 |
| Density (calculated) | 1.524 mg/m3 |
| Absorption coefficient | 0.984 mm-1 |
| F(000) | 1104 |
| Crystal size | 0.27 × 0.10 × 0.05 mm3 |
| Theta range for data collection | 2.49 to 30.31° |
| Index ranges | -38 <= *h* <= 37, -11 <= *k* <= 11, -13 <= *l* <= 13 |
| Reflections collected | 20264 |
| Independent reflections | 5901 [*R*(int) = 0.0420] |
| Completeness to theta = 25.00 ° | 99.9% |
| Absorption correction | Semi-empirical |
| Max. and min. transmission | 0.9524 and 0.7769 |
| Refinement method | Full-matrix least-squares on *F*2 |
| Data / restraints / parameters | 5901 / 98 / 308 |
| Goodness-of-fit on *F*2 | 1.126 |
| Final *R* indices [I > 2sigma (*I*)] | *R*1 = 0.0477, *wR*2 = 0.1005 |
| *R* indices (all data) | *R*1 = 0.0611, *wR*2 = 0.1042 |
| Absolute structure parameter | 0.039 (16) |
| Largest diff. peak and hole | 0.556 and -0.921 e.Å-3 |

**Fugure S1.** [(TPA)Zn(OH_2_)](ClO_4_), [(TPA)Ni(OH_2_)](ClO_4_), [(TPA)Cu(OH_2_)](ClO_4_)

Top: [(TPA)Zn(OH_2_)](ClO_4_) (**1**), Middle: [(TPA)Ni(OH_2_)](ClO_4_) (**2**), Bottom: [(TPA)Cu(OH_2_)](ClO_4_) (**3**)

**Table S2. Absorbance change of TPAM-OH_2_**

| Complexes | *A*_0_ (×10^2^) | *A*_e_ | *A*_0_-*A*_e_ | d(ln(*A*’-*A*_e_))/d*t* | -*Q* factor | *v*_int_ | *k*_obs_ |
| --- | --- | --- | --- | --- | --- | --- | --- |
| L Zn -OH _2_ 1 TPAZn-OH_2_ 1 | 0.450445.04 ×10^2^ | 0.04514.51 ×10^2^ | 0.405340.53 ×10^2^ | -0.1821-18.21 ×10^2^ | -0.0734-7.34 ×10^2^ | 0.00541885.42 ×10^3^ | 641.2734641.27 |
| LZn-OH _2_ 2 TPAZn-OH_2_ 2 | 0.449544.95 ×10^2^ | 0.04674.67 ×10^2^ | 0.402740.27 ×10^2^ | -0.1815-18.15 ×10^2^ | -0.0734-7.34 ×10^2^ | 0.00536465.36 ×10^3^ | 634.8676634.87 |
| LZn-OH _2_ 3 TPAZn-OH_2_ 3 | 0.504050.40 ×10^2^ | 0.04644.64 ×10^2^ | 0.457545.75 ×10^2^ | -0.1687-16.87 ×10^2^ | -0.0734-7.34 ×10^2^ | 0.00566665.67 ×10^3^ | 670.6000670.60 |
| LZn-OH _2_ 4 TPAZn-OH_2_ 4 | 0.449644.96 ×10^2^ | 0.04714.71 ×10^2^ | 0.402540.25 ×10^2^ | -0.1820-18.20 ×10^2^ | -0.0734-7.34 ×10^2^ | 0.005375025.38 ×10^3^ | 636.0973636.10 |
| LNi-OH _2_ 1 TPANi-OH_2_ 1 | 0.466046.60 ×10^2^ | 0.05475.47 ×10^2^ | 0.411341.13 ×10^2^ | -0.1432-14.32 ×10^2^ | -0.0734-7.34 ×10^2^ | 0.004321524.32 ×10^3^ | 511.4226511.42 |
| LNi-OH _2_ 2 TPANi-OH_2_ 2 | 0.462246.22 ×10^2^ | 0.05245.24 ×10^2^ | 0.409840.98 ×10^2^ | -0.1401-14.01 ×10^2^ | -0.0734-7.34 ×10^2^ | 0.004214974.21 ×10^3^ | 498.8126498.81 |
| LNi-OH _2_ 3 TPANi-OH_2_ 3 | 0.473947.39 ×10^2^ | 0.05295.29 ×10^2^ | 0.420942.09 ×10^2^ | -0.1603-16.03 ×10^2^ | -0.0734-7.34 ×10^2^ | 0.004953604.95 ×10^3^ | 586.2249586.22 |
| LNi-OH _2_ 4 TPANi-OH_2_ 4 | 0.465346.53 ×10^2^ | 0.05235.23 ×10^2^ | 0.412941.29 ×10^2^ | -0.1420-14.20 ×10^2^ | -0.0734-7.34 ×10^2^ | 0.004303414.30 ×10^3^ | 509.2788509.28 |
| LCu-OH _2_ 1 TPACu-OH_2_ 1 | 0.469246.92 ×10^2^ | 0.08258.25 ×10^2^ | 0.386638.66 ×10^2^ | -0.1597-15.97 ×10^2^ | -0.0734-7.34 ×10^2^ | 0.004530644.53 ×10^3^ | 536.1702536.17 |
| LCu-OH _2_ 2 TPACu-OH_2_ 2 | 0.469846.98 ×10^2^ | 0.08508.50 ×10^2^ | 0.384838.48 ×10^2^ | -0.1619-16.19 ×10^2^ | -0.0734-7.34 ×10^2^ | 0.004573424.57 ×10^3^ | 541.2337541.23 |
| LCu-OH _2_ 3 TPACu-OH_2_ 3 | 0.469046.90 ×10^2^ | 0.08178.17 ×10^2^ | 0.386938.69 ×10^2^ | -0.1617-16.17 ×10^2^ | -0.0734-7.34 ×10^2^ | 0.004592844.59 ×10^3^ | 543.53071543.53 |
| LCu-OH _2_ 4 TPACu-OH_2_ 4 | 0.467746.77 ×10^2^ | 0.07887.88 ×10^2^ | 0.388938.89 ×10^2^ | -0.1623-16.23 ×10^2^ | -0.0734-7.34 ×10^2^ | 0.004631604.63 ×10^3^ | 548.11818548.12 |

**Figure S2. Conversion between observed (*k*_obs_) and pH-independent (*k*_ind_) rates**

The total concentration of the catalyst is [M_tot_] = [M-OH^-^] + [M-OH_2_]. The observed rate constant *k*_obs_ can be calculated from

*v*_init_ = *k*_obs_[M_tot_][CO_2_] (1)

where *v*_init_ is measured initial velocity. In terms of the pH-independent rate constant *k*_ind_,

*v*_init_ = *k*_ind_[M-OH^-^][CO_2_]. (2)

Combining the two equations yields

| *k*_ind_ = | [M_tot_] | = *k*_obs_ (1 + 10^pKa-pH^) (3) |
| --- | --- | --- |
|  | [M-OH^-^] |  |
